# Homœopathic Victim

**Published:** 1850-01

**Authors:** 


*Homœopathic Victim.*—The Countess of Blessington, whose misfortunes drove her from Great Britian, died very lately in Paris, of apoplexy. The unfortunate lady was chiefly under the guidance of the homœopathic quacks, and Mr. Simon, a homœopathic doctor, was summoned to her *assistance* (?) in her fatal illness. The quack stood by her bedside, and pronounced her disease to be apoplexy! For this malady, of course, homœopathy had no remedy, no treatment.

Such events bring this absurd form of quackery to the true and severe test. All must see the perfect impotency of an infinitesimal dose against a ruptured blood vessel within the cerebrum! What can a globule do with a clot of blood among the fibres of the brain? Occurrences of this kind ought to prove a lesson and a warning to our nobility. Such cases as those of Sir Francis Burdett; Lady Denbigh, who died of uterine hemorrhage, homœopathically; and the present case of Lady Blessington, speak louder against the fashionable quackeries than any homily of orthodox medicine.—*Lancet.*

